# Compact Metasurface-Based Optical Pulse-Shaping Device

**DOI:** 10.1021/acs.nanolett.2c04980

**Published:** 2023-04-17

**Authors:** René Geromel, Philip Georgi, Maximilian Protte, Shiwei Lei, Tim Bartley, Lingling Huang, Thomas Zentgraf

**Affiliations:** † Department of Physics, Paderborn University, Warburger Strasse 100, D-33098 Paderborn, Germany; ‡ Institute for Photonic Quantum Systems (PhoQS), Paderborn University, Warburger Strasse 100, D-33098 Paderborn, Germany; § School of Optics and Photonics, Beijing Institute of Technology, 100081, Beijing, China; ∥ Kunming Institute of Physics Key Laboratory of Low-light Night Vision Technology, Xi’an 710065, China

**Keywords:** nanophotonics, optical pulse-shaping, plasmonic metasurface, SHG-FROG, dispersion

## Abstract

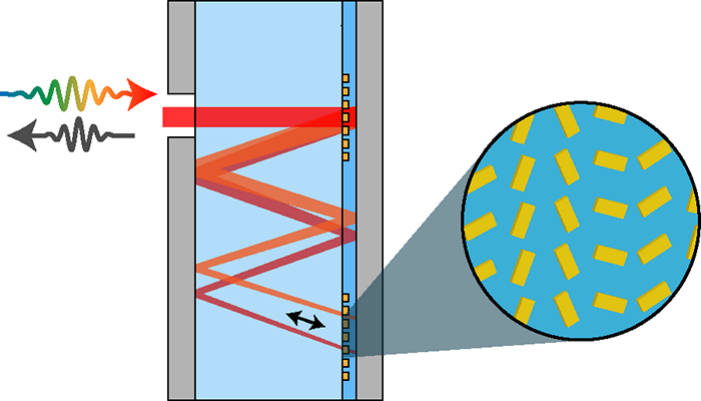

Dispersion is present in every optical setup and is often an undesired effect, especially in nonlinear-optical experiments where ultrashort laser pulses are needed. Typically, bulky pulse compressors consisting of gratings or prisms are used to address this issue by precompensating the dispersion of the optical components. However, these devices are only able to compensate for a part of the dispersion (second-order dispersion). Here, we present a compact pulse-shaping device that uses plasmonic metasurfaces to apply an arbitrarily designed spectral phase delay allowing for a full dispersion control. Furthermore, with specific phase encodings, this device can be used to temporally reshape the incident laser pulses into more complex pulse forms such as a double pulse. We verify the performance of our device by using an SHG-FROG measurement setup together with a retrieval algorithm to extract the dispersion that our device applies to an incident laser pulse.

Femtosecond laser pulses are a vital part of pump–probe experiments and in nonlinear optical applications given that their high peak power results in near-instantaneous excitations and strong nonlinear interactions. However, the propagation of a short optical pulse through optical components like lenses or beamsplitters introduces dispersion which is ultimately broadening the laser pulse and decreasing the obtainable peak power. This effect is especially critical for ultrashort laser pulses with sub-100 fs pulse durations considering their large spectral bandwidth. To ensure that bandwidth-limited pulses arrive at the desired point of application, pulse compressors consisting of gratings and prisms are commonly used for precompensating the accumulated optical phase dispersion.^1^ However, these devices are limited to correcting only second-order dispersion while adding additional higher-order dispersion to the laser pulses.

A less restrictive way of compensating dispersion is the use of spectral pulse shaping devices, which are often realized with spatial light modulators (SLMs). SLMs are liquid crystal based reprogrammable pixel arrays with modulation speeds of several kHz that can locally alter the phase or intensity of incident light. They are used in a variety of optical applications,^2,3^ where they can act as lenses, gratings, or holograms. With the additional degrees of freedom inherently available in an SLM, not only can one compensate for higher-order dispersion, one can in fact modify the pulse shape arbitrarily.^4^ Such SLM-based pulse-shaping devices usually contain a grating and a focusing mirror to spatially disperse the spectral information before its interaction with the SLM. Mansuryan et al. proposed a more compact design using only a lens and a phase-only SLM that is programmed as a swept blazed grating in Littrow configuration that generates spatial dispersion in combination with additional phase information.^5^ Common to all the mentioned approaches is that they use free-space propagation over a range of at least several centimeters making it difficult for integrating such concepts of pulse shaping into microoptical or integrated optical devices. Here, we propose using optical metasurfaces for building ultracompact pulse-shaping devices that have the potential for being integrated into miniaturized optical setups.

Metasurfaces are artificial, optically thin structures exhibiting properties that do not just result from the chosen material of the system but also from its geometry.^6^ More specifically, the nanostructures of a metasurface, which are usually arranged in a periodic subwavelength lattice, can be tailored to control the local optical response. Similar to SLMs, they allow for high control of light by precisely tailoring its local phase and intensity by exciting localized modes in plasmonic or dielectric nanostructures.

In recent years, a plethora of metasurface applications was demonstrated such as meta-lenses with high numerical aperture or diffraction-limited focal points for imaging,^7−11^ multiplexed linear and nonlinear holography^12−15^ as well as bandpass filters^16^ and waveplates.^17,18^ Their subwavelength size allows for compact and flat structures, utilized in on-chip photonic devices.^19−21^ In the recent work by Faraji-Dana et al., the authors demonstrated a compact metasurface-based spectrometer that uses multiple metasurfaces to spatially separate the spectral information and focus it onto a detector chip array.^22^ For this device, wavelength resolutions of up to 1.25 nm have been reported, whereas higher resolution typically comes at the cost of increased propagation distances within the device making it less compact. Furthermore, metasurfaces have also been used in several free-space pulse shaping applications where they are often replacing traditional SLMs.^23,24^ However, there the spectral pulse components are spatially separated and focused with conventional optical components like gratings and lenses, resulting in a large overall device size.

Recently, Ossiander et al. showed pulse compression with a single metasurface that relied on anomalous dispersion introduced by mode engineering. However, this device does not allow the tailoring of higher-order dispersion, and its compensation scales with the metasurface thickness.^25^

In this work, we combine the ideas of using the metasurface for spectral separation and spectral phase manipulation to realize a compact metasurface-based pulse-shaping device. The device consists of two plasmonic metasurfaces between two parallel silver mirrors that arbitrarily alter the spectral phase of femtosecond laser pulses on a volume of less than 2 mm^3^. To realize such a functionality, an off-axis-focusing metalens that introduces angular dispersion in combination with a grating-metasurface encoded with appropriate spectral phase information is fabricated on a regular glass slide substrate.

A schematic illustration of the metasurface pulse-shaping device is shown in Figure 1. The two parallel silver mirrors enable a successive interaction of the laser light with the metasurfaces MS1 and MS2 while simultaneously enlarging the effective propagation length for an increased spectral resolution. Both metasurfaces are fabricated on one side of a 2 mm-thick BK7 substrate, leading to a compact design. The metasurfaces themselves consist of rectangular-shaped plasmonic meta-atoms which encode a Pancharatanam–Berry phase via their orientation angle, allowing for a broadband phase control for the circular polarization states in reflection.^26,27^ MS1 is a circular-shaped metasurface field with a 400 μm diameter. It incorporates a superposition of a phase gradient (effective grating period of 2400 nm) resulting in a diffraction angle of θ = 12.1° at a wavelength of 760 nm and a convex lens phase profile. The former introduces angular dispersion and the latter focuses the spectrally separated light on MS2 resulting in an extended focus of the beam on MS2 along one direction and an expected small beam size in the other, comparable to a cylindrical lens. Because of this, MS2 is a rectangular-shaped metasurface field (400 μm × 100 μm), which incorporates twice the phase gradient of MS1 in the opposite direction to relocate all spectral components back to MS1. There, a second interaction with MS1 collimates all wavelengths into a single beam which leaves the device back through the input aperture. All mentioned phase profiles encoded on MS1 and MS2 are shown in Figure 2. In addition, MS2 can also contain wavelength-dependent phase information required for the pulse shaping as the laser pulse is spectrally separated on this metasurface.

**Figure 1:**
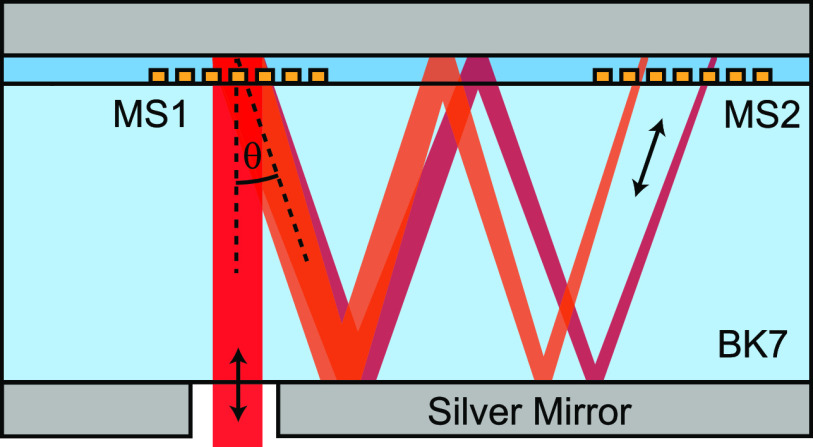
Schematic illustration of the pulse shaping device. Two parallel silver mirrors with an aperture facilitate a beam interaction with two reflective metasurfaces (MS1 and MS2) on the same BK7 substrate. MS1 acts as a lens and a grating (diffraction angle θ for center wavelength), introducing angular dispersion and focusing the incident laser beam on MS2. In addition to the wavelength dependent phase information, MS2 acts as a grating with twice the phase gradient of MS1, sending the beam back to the first metasurface. On MS1, the beam is then collimated and diffracted normal to the surface.

**Figure 2:**
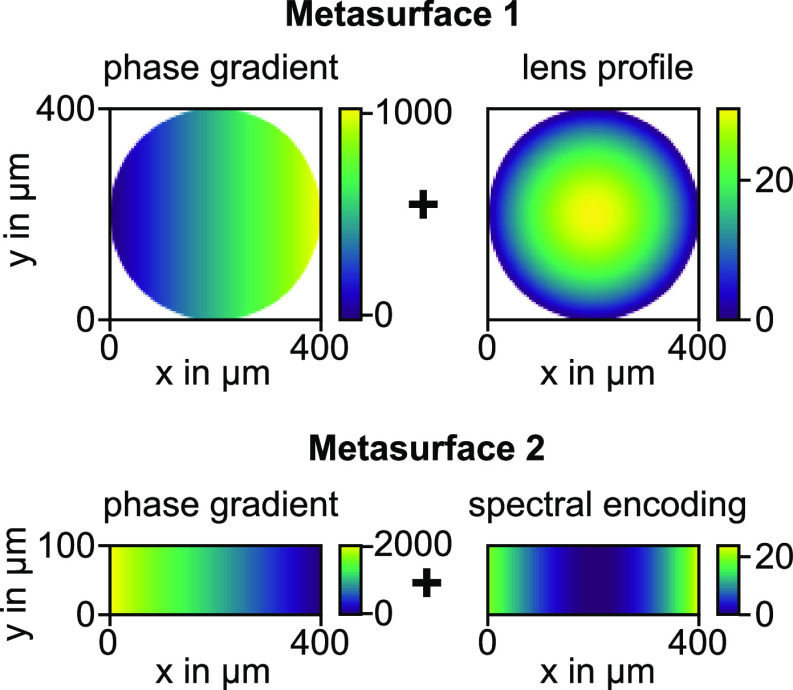
Phase information encoded on MS1 and MS2 used for the pulse shaping functionality. Both metasurface fields facilitate a phase gradient with opposite orientations. On top of these phase gradients a second phase profile is superimposed in both fields: On MS1, the additional phase is a convex lens phase profile, and on MS2, the additional phase is a spectral phase encoding that can be used to alter the dispersion. The spectral phase encoding shown here compensates for the inherent dispersion of the device (all phases are given in rad).

Note that the pulse shaping device inherently introduces dispersion due to the wavelength-dependent refractive index of the BK7 glass and the spectrally different geometrical beam path lengths within the device. For preserving the temporal pulse shape after passing through the device, this dispersion has to be compensated by MS2. Furthermore, an additional spectral phase encoding can be introduced on MS2 to further modify the pulse shape analogous to an SLM-pulse shaper.

For our experimental studies, we use pulses with a pulse length of 45 fs at 760 nm center wavelength resulting in a spectral bandwidth of 20 nm as well as an additional prism pulse compressor to alter the second-order dispersion of the laser pulse. A λ/4-plate generates right circular polarized light while also transforming the device’s output back to a linear polarization state. To characterize the temporal pulse shape and its dispersion, SHG-FROG (second harmonic generation–frequency-resolved optical gating) measurements are done using an intensity autocorrelator (GECO from Light Conversion) and a fiber-coupled spectrometer (OceanOptics). During an SHG-FROG measurement, a spectrogram (“FROG trace”) is recorded, which is a spectrally resolved autocorrelation of the measured pulse. In conjunction with a retrieval algorithm, the original pulse shape can be derived from this trace within its ambiguity.^26,28−30^

It is worth mentioning that an interaction with our reflective geometrical phase metasurface generates a circular polarization of the same helicity whereas an ordinary mirror interface reflection changes the helicity. Since the number of interface reflections is odd due to the symmetric nature of our device, the overall output signal has the same helicity as the incident beam. That has the advantage that any unwanted light which is directly reflected back by the entrance aperture or MS1 will undergo a polarization change to the opposite helicity and can be easily suppressed with linear polarization filtering after passing the waveplate again. A detailed description of the experimental setup can be found in the Supporting Information.

For our experimental demonstration, we fabricated and measured four different sets of MS1 and MS2 with different functionalities (see Figure 3). In the first case, MS2 exhibits no phase encodings besides the base gradient profile for the deflection. Hence, the pulse is broadened after passing through the pulse-shaping device. In the second case, the dispersion of the device itself is compensated so that the temporal pulse profile is not changed by the propagation through the device. The associated spectral phase encoding has a parabolic shape, which already indicates that most of the compensated device dispersion belongs to the second order, which is commonly called group delay dispersion (GDD). Meanwhile, higher-order dispersion terms like third-order dispersion (TOD) are only weakly contributing. To demonstrate that the TOD can be altered as well, the third case does not only contain the compensation phase encoding but also a large 30000 fs^3^ TOD encoding on top, which should notably change the shape of the FROG trace. In the last case, we altered the spectral phase such that a double pulse with a temporal separation of 200 fs is generated. Ideally, this would not just involve a change of the spectral phase but also of the spectral amplitude. However, in good approximation, we can also achieve the same effect by applying a periodic π phase shift to the laser spectrum for 2.5 THz intervals on top of the standard phase compensation term. Furthermore, MS2 could be also used to introduce additional group delay (GD) for the pulse. However, the encoding of additional GD on MS2 besides the already present GD by the diffraction grating requires a large phase gradient which is ultimately limited by the resolution provided by the unit cell periodicity.

**Figure 3:**
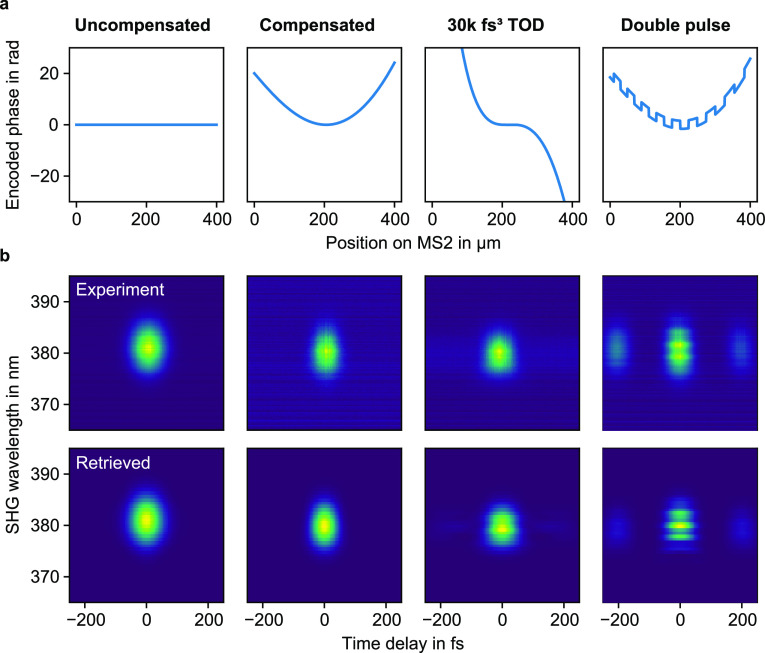
(a) Spectral phase information encoded in different MS2 to achieve distinct functionalities. The first design contains no further phase information (“uncompensated”), whereas the other three designs are meant to compensate the inherent device dispersion (“compensated”), create an additional TOD of 30000 fs^3^ (“30k fs^3^ TOD”), and generate a double pulse with a temporal delay of 200 fs (“double pulse”). Note that the latter two phase encodings also include the device dispersion compensation. (b) Measured (top row) and retrieved (bottom row) SHG-FROG traces for the different functionalities plotted as SHG signal strength over SHG wavelength and time delay.

For all four device configurations as well as for the original pulse, we measured the SHG-FROG traces and retrieved the temporal pulse shapes (for details see the Supporting Information). To analyze the device behavior more rigorously, we performed these measurements not just once, but multiple times with varying initial pulse dispersions that were controlled at the integrated pulse compressor in our light source. In Figure 3, the four measured traces are plotted for the pulse compressor position, where the trace width along the time delay axis is the smallest in the reference measurement where the laser pulse is only reflected by a mirror instead of passing through the device (corresponding to position 480 in Figure 4a). For this setting, the original pulse should have close to zero GDD, since the second-order dispersion tends to broaden a pulse in the time domain and its autocorrelation, which is depicted in the FROG trace.

**Figure 4:**
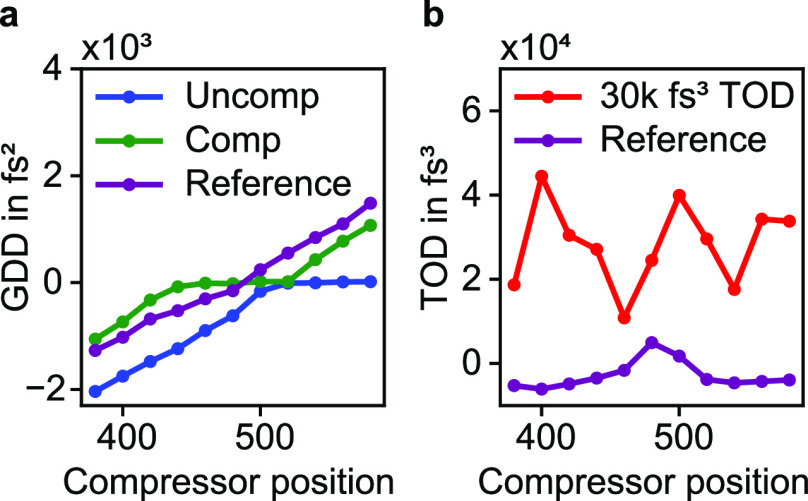
(a) Measured second-order dispersion of the uncompensated and compensated pulse shaping device in comparison to the original laser pulse (reference) for different pulse compressor settings. Here one compressor step should roughly correspond to a change in GDD of 12.5 fs^2^ and in TOD of 8.3 fs^3^. Ideally, the compensated MS2 design should have the same dispersion as the reference, while the uncompensated device is supposed to accumulate a GDD of −784 fs^2^. Note that due to experimental uncertainties an unphysical trace form can be measured, which violates the Fourier-based limitations. After the retrieval, the closest physical trace is assumed which might explain the constant GDD value of zero over a certain range of the compressor steps in two of the three cases. (b) The used SHG-FROG setup barely allows a sensible retrieval of TOD. However, for a very strong TOD encoding of 30k fs^3^, a clear deviation from the reference pulse can be observed.

If we look at the measured SHG-FROG traces for the compensated and the uncompensated pulse shaper (Figure 3b), we can see in both cases the expected Gaussian distribution. However, the trace for the uncompensated device is slightly wider along the time delay axis, which can be explained by the inherent dispersion of the device. As for the 30k fs^3^ TOD design, an hourglass shape in the trace was expected. However, in the measured trace this is barely the case as it has more of a triangular shape with the horizontal side along the smaller wavelengths. This can be explained by a small GDD contribution, which has the same (positive) sign as the encoded TOD. For the last case, a double pulse should generate three peaks in the trace along the time delay axis and also exhibit a characteristic interference pattern for the middle spot along the spectral axis which can be seen in the measured trace. Here the middle spot results from the self-interaction of the two pulses, while the outer spots result from the cross-interaction.

If we compare the retrieved traces with the measured traces, we can observe that they qualitatively match. However, there are still some differences caused on the one hand by the applied postprocessing (including background correction, noise canceling, centering, and rescaling) as well as the physical constraints introduced in the retrieval algorithm itself (more details on the postprocessing of the traces can be found in the Supporting Information).

In the next step, we studied the dispersion changes that were introduced by our pulse shapers. For that, we fitted a third-order polynomial to the spectral phase distribution of all retrieved pulses, which allowed us to deduct the second and third-order dispersion of the pulse. The retrieved GDD and TOD values are plotted in Figure 4 over the prism position of the external pulse compressor of our laser system (larger values of the prism position mean larger GDD is introduced to the pulses). Note that the SHG-FROG trace is ambiguous in terms of the sign of the dispersion, which were determined by the physical context. From the retrieved dispersion plots, we conclude that the device with the compensated GDD has approximately the same second-order dispersion as the reference pulse, while the GDD of the uncompensated case is considerably lower. However, the ratio between the width and height of the traces seems to be systematically slightly off for the measurements with the pulse shaper. This leads on the one hand to unphysical offsets in the retrieved GDD values and on the other hand to a plateau at zero over several pulse compressor steps. Despite these errors, we are still able to verify the applied second-order dispersion by averaging the change in GDD with regards to the reference measurement over all compressor positions, which leads to a GDD change of (−767 ± 629) fs^2^ for the uncompensated case and of (−17 ± 768) fs^2^ for the compensated pulse shaper. The former value is quite close to the calculated inherent device dispersion of −784 fs^2^ for a wavelength of 760 nm. As for the TOD evaluation, the data shows a large standard deviation across different pulse compressor positions. However, the order of magnitude of the TOD can be extracted from the mean value relative to the reference measurements, which leads to a value of (31 ± 22) × 10^3^ fs^3^ and is therefore close to the encoded TOD of 30000 fs^3^.

To further analyze the device’s performance for the generation of the double pulse, we also looked at the retrieved pulse in the time domain (Figure 5). Here, the two individual pulses have a separation of 207 fs while the two peaks differ in their electric field amplitude significantly, which was not intended by our design. This difference can be explained by the alternating phase pattern for the double pulse on MS2 which is shown in Figure 3a. Considering that the laser pulse is only covering a width on MS2 of less than 100 μm, a nonequal illumination of the 0 and π levels in the phase encoding can result in an unbalanced power distribution, which leads to uneven pulse amplitudes in the time domain. Nevertheless, this demonstration shows that our device can be used for pulse-shaping applications beyond the standard dispersion compensation.

**Figure 5:**
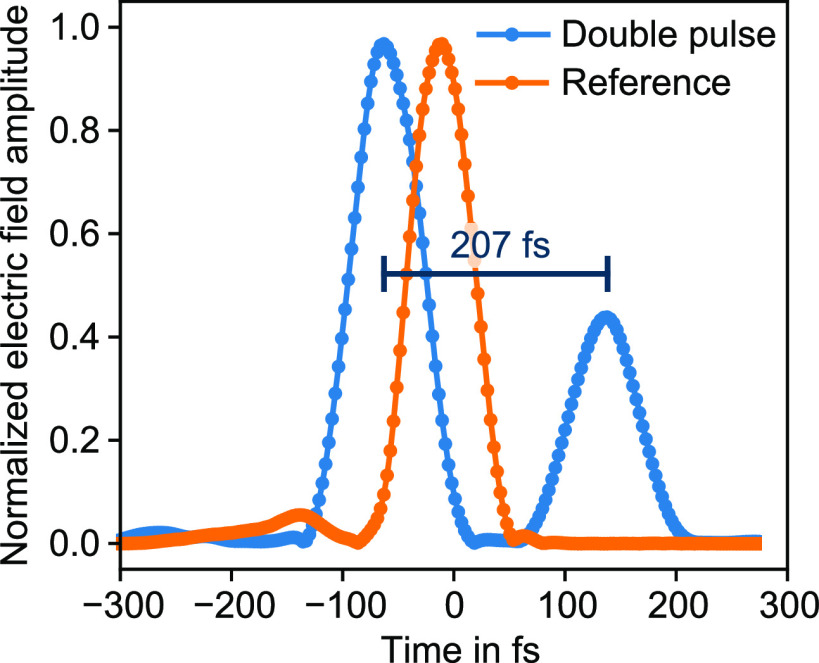
Retrieved electric field amplitude of the generated double pulse plotted over time. Note that the SHG-FROG retrieval cannot differentiate whether the second pulse is advanced or retarded relative to the original pulse. However, the designed temporal separation of 200 fs can be verified from this retrieval.

In this work, we demonstrate a compact pulse-shaping device that allows the interaction of a laser beam with two metasurfaces within a single glass substrate. Our device can apply an arbitrarily designed spectral phase dispersion to an incident laser pulse for temporal pulse shaping. We show that the concept can be used for more complex pulse-shaping applications like the generation of a double pulse train. The design freedom in the spectral phase allows the compensation of the inherent second-order dispersion that is introduced by the device itself. Experimentally, we verified the dispersion of the laser beam by using SHG-FROG measurements. The utilized retrieval algorithm also allows for a reliable retrieval of GDD from our measured traces when large second-order dispersion was present. We also demonstrated that higher-order dispersion terms can be independently tailored at the example of large values for the TOD. Furthermore, the spectral resolution of our device can be enhanced by increasing the number of internal reflections or by increasing the angular dispersion through a smaller grating period on MS1.

One shortcoming of the design is the use of plasmonic metasurfaces for the spatiotemporal shaping of the pulses, which limits the beam power because of the low damage threshold and results in the low total efficiency of the device. Using dielectric metasurfaces with lower loss and higher damage threshold might circumvent these limitations and increase the overall performance of our device.

Our metasurface-based pulse-shaping device can be used in space-limited applications, where the dispersion of pulses might be important. Here, we present a pulse shaping device working in reflection whereas a device operating in transmission could also be realized by placing metasurfaces on both sides of the glass substrate. Such a design can increase the device output since the experimental setup would not need a beamsplitter. The potential to tailor the dispersion beyond traditional prism- or grating-based pulse compressors makes them also interesting for complex pulse-shaping applications like in quantum optics where particular temporal pulse shapes are highly desired.^31^ Furthermore, the obtained temporal pulse shaping can be combined with spatial pulse shaping at the same metasurface to increase the pulse complexity even further.

## Data Availability

Data supporting this study are openly available from Zenodo at DOI 10.5281/zenodo.7467230.

## Supporting Information Available

Description of antenna design via RCWA simulation, description of sample fabrication, experimental setup, and discussion of device efficiency, postprocessing of measured SHG-FROG traces, retrieval algorithm of these traces, and measured SHG-FROG traces (PDF)
