# Supplementary material for: Compact Metasurface-Based Optical Pulse-Shaping Device
Source: Nano Lett. 2023 Apr 17;23(8):3196–201. doi: 10.1021/acs.nanolett.2c04980 (PMC10143620; doi:10.1021/acs.nanolett.2c04980)
Supplement: Supplementary file 1 — nl2c04980_si_001.pdf [file nl2c04980_si_001.pdf]

Compact metasurface-based optical pulse-shaping device

René Geromel[1,2§], Philip Georgi[1,2§], Maximilian Protte[1,2], Shiwei Lei[3,4], Tim Bartley[1,2], Lingling Huang[3], and Thomas Zentgraf[1,2]

[1] Department of Physics, Paderborn University, Warburger Str. 100, D-33098 Paderborn, Germany
[2] Institute for Photonic Quantum Systems (PhoQS), Paderborn University, Warburger Str. 100, D-33098 Paderborn, Germany
[3] School of Optics and Photonics, Beijing Institute of Technology, 100081, Beijing, China
[4] Kunming Institute of Physics Key Laboratory of Low-light Night Vision Technology, Xi'an 710065, China

[§] R.G. and P.G. contributed equally to this paper.

1. Metasurface design and phase encodings

Since our metasurface design is based on the Pancharatnam-Berry phase, it was sufficient to optimize one structure in a periodic environment for a maximal polarization conversion in the circular polarization states. In our optimized structure with a periodicity of 300 nm, gold nanoantennas with the size of 220 nm x 95 nm and a height of 30 nm are placed 130 nm away from the silver mirror. The associated unit cell is shown in Figure S1a and the utilized refractive indices for gold and silver were obtained from [1] and the Sellmeier coefficients for the BK7 glass substrate were taken from the supplementary material of [2]. In the numerical simulations, we observed that the metasurface design is insensitive to parameter changes and works broadband with efficiencies above 80% (see Figure S1b). However, considering that light has to perform multiple reflections, both on the metasurface and the silver mirrors in the pulse-shaping device, we expected a significant power loss for the whole device in the order of 50% of the incident power.

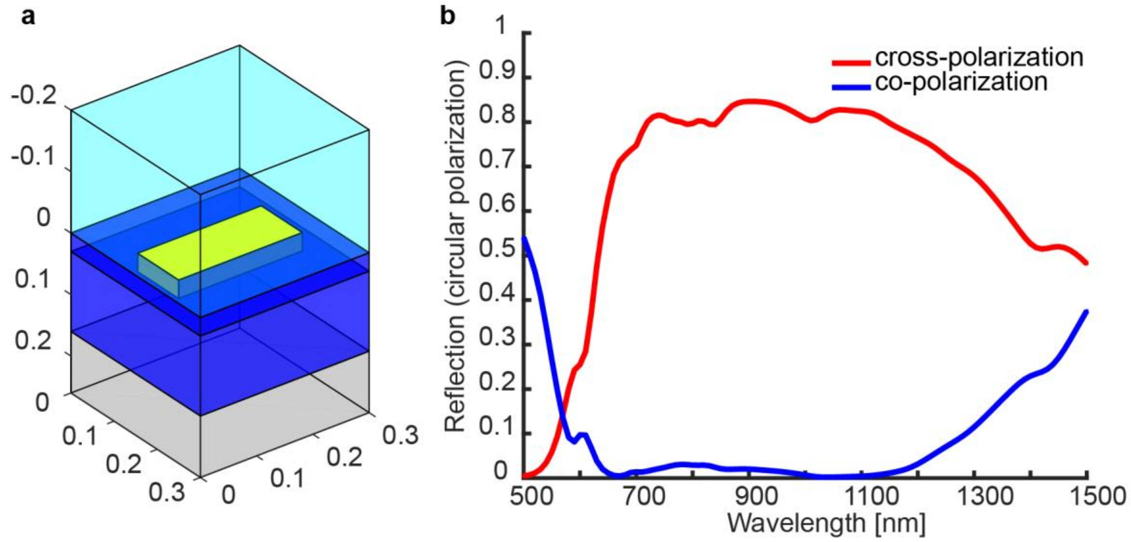

Figure S1: Rigorous coupled wave analysis (RCWA) for the metasurface unit cell. a) Plot of the unit cell in the RCWA simulation. In the top layer is BK7 glass, followed by the embedded gold antenna. Below is the spacer layer of spin-on glass as well as the silver backplane. The space coordinates are given in µm. b) Reflection of the metasurface for illumination from the glass side with circularly polarized light. The used antenna structure works broadband since the cross-polarization reflection, which is equivalent to structure efficiency, stays above 80% over a large spectral domain

from 700 nm - 1100 nm. For the used wavelength of 760 nm in the experiment, we calculated a cross-polarization reflection of 80.6 % and a co-reflection of 2.7 %.

The second step for the metasurface design is the phase encoding. The first metasurface (MS1) encodes a superposition of a phase gradient and a lens profile:

$$\phi_{MS1}(x,y) = \phi_{grad1}(x) + \phi_{lens}(x,y)$$

Both can be calculated analytically via:

$$\phi_{grad1}(x) = \frac{2\pi x}{P}$$

$$\phi_{lens}(x) = \frac{2\pi \cdot n_{BK7}(\lambda_{0,c})}{\lambda_{0,c}} \cdot \left(f - \sqrt{f^2 + x^2 + y^2}\right)$$

Here $P = 2400$ nm is the periodicity of the gradient structure, n_{BK7} is the refractive index of BK7 and $\lambda_{0,\mathrm{c}} = 760$ nm is the central wavelength. Meanwhile, the focal length f is equal to the path length from the center of MS1 to MS2 for the central wavelength.

The second metasurface (MS2) encodes the superposition of a phase gradient and a spectral phase:

$$\phi_{MS2}(x,y) = \phi_{grad2}(x) + \phi_{spectral}(\omega(x))$$

The phase gradient can be calculated via:

$$\phi_{grad2}(x) = -\frac{4\pi x}{P}$$

Meanwhile, for the spectral phase we calculate the phase acquisition for the propagation of the device, which can be estimated as:

$$\phi_{device}(\omega) = \left(2 \cdot \left(d + s(\omega)\right) \cdot n_{BK7}(\omega)\right) \cdot \frac{\omega}{c_0}$$

Here, $d = 2$ mm is the thickness of the device, while $s(\omega)$ is the propagation length from MS1 to MS2. We obtain now the four presented phase encodings for MS2. The first phase encoding for the uncompensated case is trivial:

$$\phi_{unc}(\omega) = 0$$

In the compensated case, we encoded the inverse device dispersion to compensate for the accumulated dispersion during the propagation in the device itself. However, since the linear terms do not contribute to the pulse shape we exclude it with a linear fit of the device dispersion.

$$\phi_{comp}(\omega) = -\phi_{device}(\omega) + \phi_{linear\ fit}(\omega)$$

The third-order dispersion can be introduced via a third-order Taylor term around the central angular frequency ω_c:

$$\phi_{TOD}(\omega) = \phi_{comp} + \frac{\mathrm{TOD}}{6} \cdot (\omega - \omega_c)^3$$

The phase for the double-pulse device can be encoded via:

$$\phi_{double\ pulse}(\omega) = \phi_{comp} + \frac{\pi}{2} \cdot \mathrm{sgn}\left(\sin\left((\omega - \omega_c) \cdot \frac{\tau_{DP}}{2}\right)\right)$$

Here *sgn* is the sign function that returns 1 for positive values and -1 for negative values and $\tau_{DP} = 200$ fs is the encoded time delay between the two pulses.

2. Fabrication

Starting from the aperture side of the device, laser lithography with the photoresist AZ4562 is used to pattern a 450 µm x 1 cm slit aperture. After developing the resist, 2 nm of chromium as an adhesion promoter and 100 nm of silver are deposited using electron beam physical vapor deposition (EBPVD). A lift-off procedure using acetone exposes the patterned aperture and three layers of IC1-200 spin-on-glass (200 nm @3000rpm, 3000acc, 40s) are spin-coated and baked (200°C for 60s) to obtain a ~600 nm layer that protects the back side during further fabrication steps.

As for the front side, PMMA (AR-P 679.04, 270 nm @3000 rpm, 3000 acc, 60 s) and Electra 92 (AR-PC 5090.02, 40 nm @4000 rpm, 4000 acc, 40 s) are spin-coated and electron beam lithography (EBL) is used to pattern a marker system used to locate the aperture on the backside and allow precise alignment of MS1 over the aperture. An MIBK-based developer is used and 30 nm of chromium is deposited via EBPVD. After the lift-off using acetone and the location of the aperture, another EBL step with similar parameters for the metasurfaces is done and 2 nm of chromium (adhesion promoter) and 30 nm of gold are deposited via EBPVD. The lift-off is done using acetone. SEM images of one MS1 and MS2 are taken to verify the nanostructure size as well as the homogeneity of the sample achieved with this procedure (Fig. S2). After the liftoff a spacer layer of IC1-200:Butan-1-ol(5:4) solution is spin-coated (130 nm @3000 rpm, 3000 acc, 40 s) and baked (200°C for 60 s). Finally, another 100 nm silver is deposited via EBPVD to serve as the mirror. The entire process flow is shown in Fig S3.

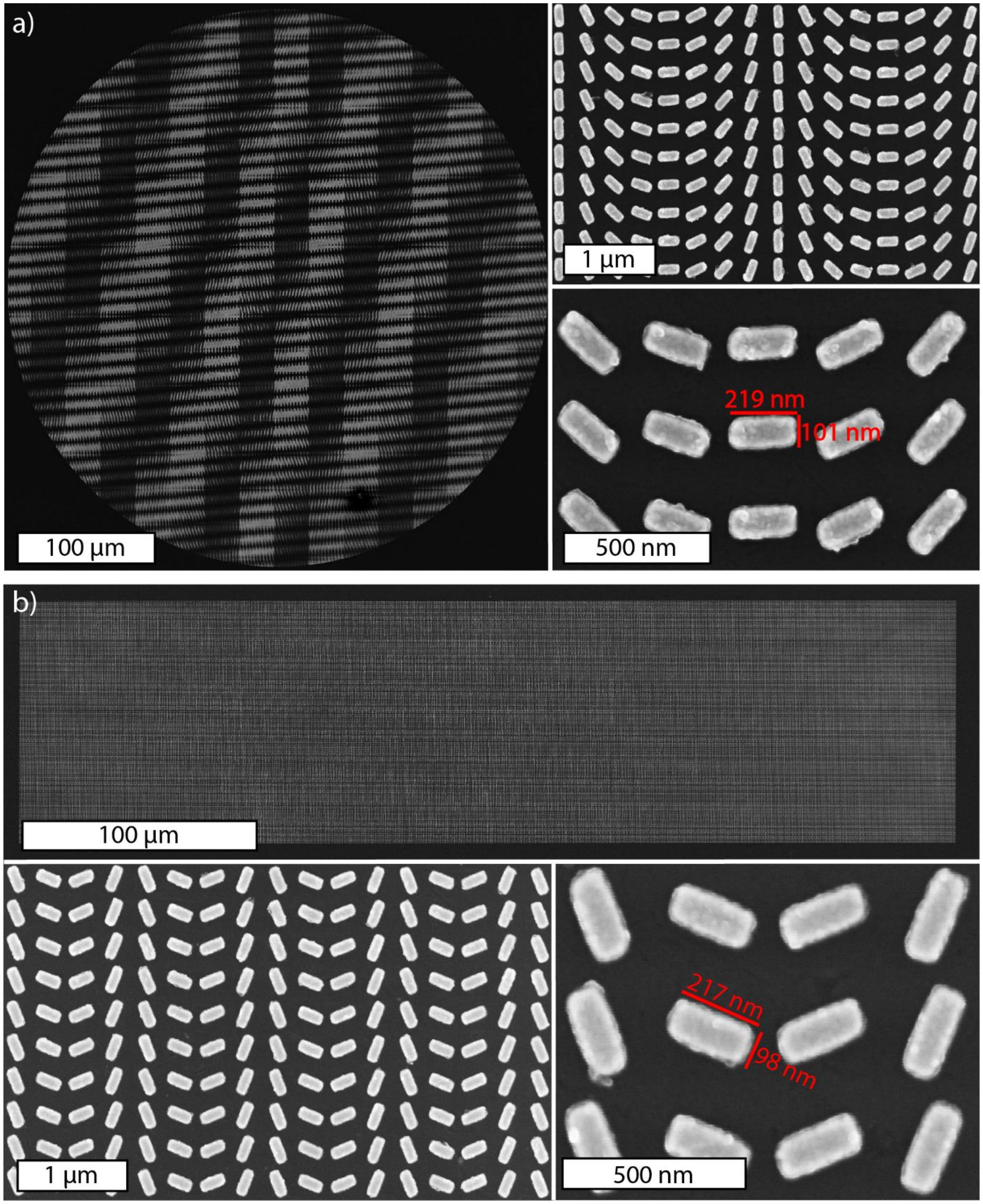

Figure S2. SEM images (topview) of a fabricated a) MS1 and b) MS2 at various magnifications including a scale bar. The fabrication matches the targeted nanostructure size of 220 nm x 95 nm for the length and width.

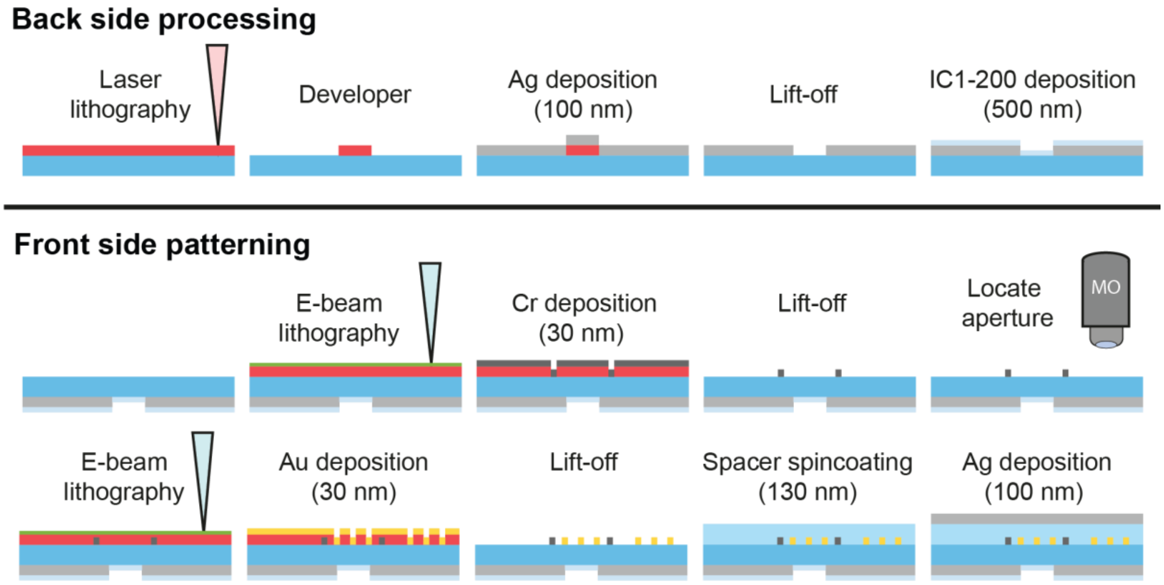

Figure S3: Fabrication of the pulse shaper sample. The aperture is fabricated using laser lithography and lift-off for the deposited silver film. Spin-on-glass (IC1-200) is spin-coated and hard-baked to create a scratch-resistant layer that protects the backside during the fabrication of the front side. On the front side, using e-beam lithography a chromium marker system is fabricated to locate the aperture on the backside and allow proper alignment of the metasurface MS1 over the aperture. The metasurfaces are fabricated similarly and a spacer layer of IC1-200 diluted with butan-1-ol in a 5:4 ratio is spin-coated to achieve a suitable spacer layer to deposit the second silver mirror.

3. Experimental setup and device efficiency

Our experimental setup uses ~45 fs laser pulses at 760 nm center wavelength with linear, horizontal polarization and a bandwidth of ~20 nm. Furthermore, an additional pulse compressor is used to adjust the second-order dispersion of the laser pulse before the experiment. Because of the focusing of the laser by MS1, MS2 is exposed to high intensity we kept the average input power below 30 mW to avoid damage of the metasurface. Since the input power from our laser system is not a limiting factor in these experiments, we opted for a 30/70 beamsplitter (R/T BS) which increases the signal that can be collected from our meta device. A lens with a high focal length (f=750 mm) is used to create a focal spot that suits the diameter of MS1 while also collimating the beam again after the device interaction. A $\lambda/4$-plate is used to generate the necessary right circular polarization (RCP) and also transforms the laser beam that interacted with the sample back to horizontal polarization whereas the part that did not interact with the metasurfaces is turned to vertical polarization. This is helpful since the nonlinear crystal (BBO) used in the autocorrelator generates polarization-dependent SHG and filters out any unwanted signal by properly setting the crystal orientation. The autocorrelator creates two beam paths via a beamsplitter and a stage to introduce a time delay τ which influences the temporal overlap of the two laser pulses on the nonlinear crystal and thus creates delay-dependent SH which is measured with a fiber-coupled spectrometer. The alignment of the sample is verified via a camera which allows the imaging of the focal spot from MS1 on the center of MS2. A schematic sketch of the experimental setup is shown in Fig S4.

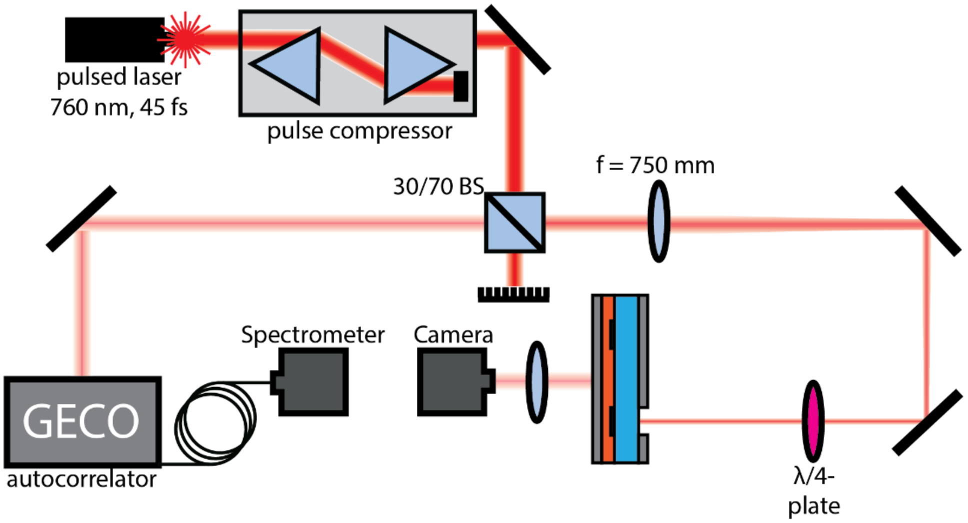

Figure S4: Illustration of the experimental setup. The setup uses a beamsplitter (BS) and a lens to focus the laser beam through the device aperture onto MS1. A λ/4-plate is used to generate right circular polarization (RCP) and an imaging setup behind the meta device allows the alignment of the sample by observing the focal plane from MS1 on MS2. The signal is then routed to an autocorrelator that is connected to a spectrometer to measure the SHG-FROG traces.

This setup also allowed to determine the conversion efficiency of the individual metasurfaces. For the first metasurface, several structures encoded with the phase gradient from MS1 containing a variation in antenna length and width without the back side mirror were fabricated and the power from the first diffraction order was compared to the incident laser power. Fig. S5 shows the measured conversion efficiencies for several MS1 designs and the parameter set that was used for the fabricated pulse shaper showed a conversion efficiency of ~50% for MS1. The efficiency of MS2 was determined in a finished pulse shaping sample that has a thinner silver mirror (30 nm) backing the metasurfaces. The method shown in Fig. S6 utilized to estimate the efficiency of MS2 makes use of the interface reflection between MS1 and MS2 and the different circular polarizations that occur at this point. When filtering the circular polarization states transmitted through the thin silver mirror, the imaging setup shown in Fig. S4 allows the comparison of the intensity from the beam that is propagating from MS1 to MS2 and vice versa. The conversion efficiency of MS2 was also determined to be ~50%. Since the optimized unit cell from Fig. S1 does not consider an application in a phase gradient structure a large deviation from the calculated conversion efficiency of 80% is observed. Further simulations using a phase gradient design for the fabricated nanostructure dimensions also yield a conversion efficiency of 52% for MS1 and MS2 for the relevant diffraction orders used in the experiment. The overall decrease comes from the large diffraction angle and an optimization with regards to the phase gradient functionality of the plasmonic nanostructures would be necessary to increase the device's efficiency. Neglecting interface reflections, the overall maximum device efficiency assuming a 50% conversion efficiency of the individual metasurface is 12.5% and since the overall device performance scales exponentially with the conversion efficiency of the single metasurface. Assuming an optimized phase gradient functionality with a conversion efficiency of 80% the overall device efficiency rises to 51% whereas for dielectric structures, which minimize the absorption losses that are typically present in plasmonic nanostructures, a conversion efficiency of 90% is a realistic value and already increases the device

performance to 73% while also overcoming possible bottlenecks with regards to low laser-induced damage thresholds.

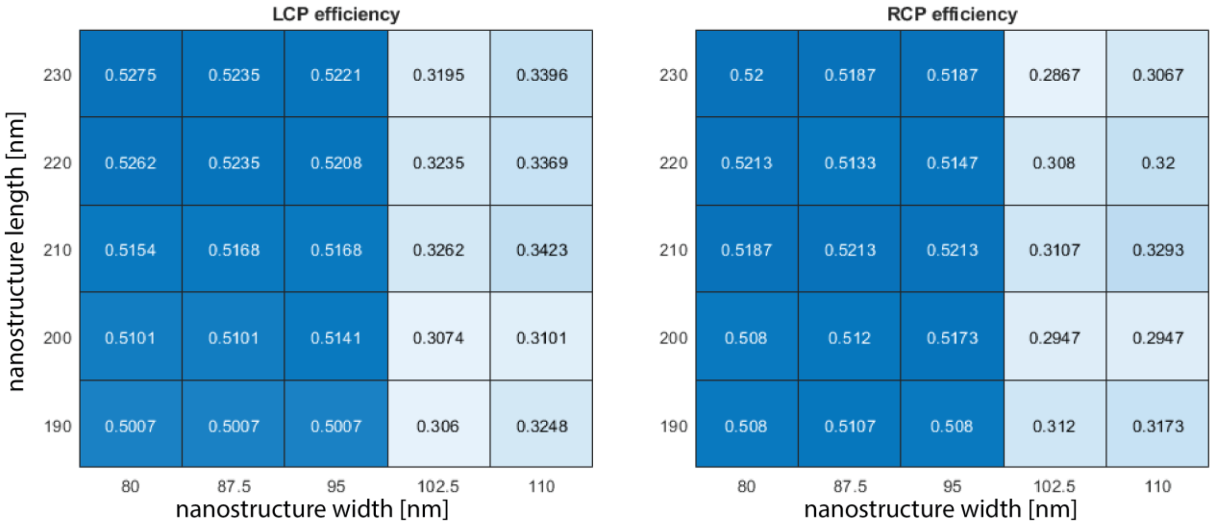

Figure S5. Measured conversion efficiency for incident LCP and RCP for a length and width variation of the nanostructures. A drop in efficiency can be observed for widths of more than 100 nm.

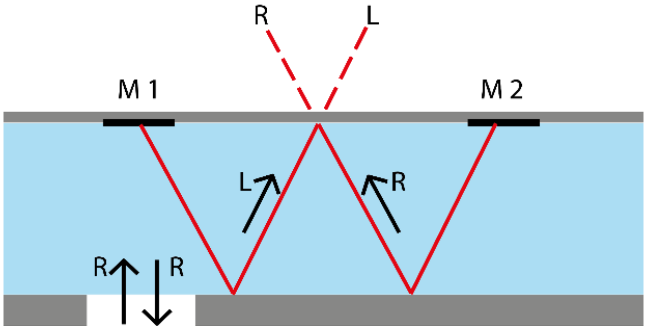

Figure S6. Illustration of the transmitted circular polarizations in the interface reflection between MS1 and MS2 used to determine the conversion efficiency of MS2 including three interface reflections. R and L refer to the helicity of the laser beam and a comparison of the transmitted LCP and RCP give a ratio of ~50%.

Regarding the diffraction efficiency, all simulations and measurements were done for the center wavelength of 760 nm, however, initial operation of our device at 800 nm center wavelength with ~60 nm bandwidth have shown similar results. The current design of MS2 addresses a wavelength range from 690 nm to 850 nm however the performance for this entire wavelength range was not tested. Additionally, as the wavelength shifts further from devices center wavelength of 760 nm a defocusing due to the wavelength dependent focusing behavior of MS1 will occur. Towards the minimum and maximum wavelength currently supported by MS2, an increase in the beam radius on MS2 of roughly 50% (from 13 µm to 18 µm) is expected while a sharp decrease in the conversion efficiency of the plasmonic nanostructure is expected below 700 nm (see Fig. S1 b)).

4. SHG-FROG trace data preprocessing

To be used in the retrieval algorithm, the FROG trace data has to be preprocessed in advance. We used four different steps in this preprocessing procedure:

1. **Smoothing:** In our measurements we found, that we had noise in the FROG Trace along the delay dimension due to laser fluctuations and along the frequency dimension presumably because of varying spectral fiber coupling efficiencies. To compensate for these effects, we applied a Savitzky-Golay filter to the frequency and the delay marginal of the trace and then applied the smoothing factors across the rows/columns of the trace.
2. **Background correction:** The ideal trace should be zero outside of the region of interest. To ensure this property, we looked at the statistics of the border values of our measured trace and subtracted the mean value and twice the standard deviation of the border region from the whole trace. Afterward, we set all negative values to zero.
3. **Delay centralization:** The ideal SHG-FROG trace is symmetric around $\tau = 0$. To correct the delay calibration, we centralized the 'center of gravity' of the measured FROG trace.
4. **Rescaling:** The resolution of the measured FROG trace is given by the spectrometer and the recorded delay steps. However, to use the trace data in the retrieval algorithm it has to be resolved in a grid, that is suitable for the fast Fourier transformation (FFT). This grid is fully determined by spectral resolution Δf and time resolution Δt. Given these two quantities, the used trace has $n \times n$ points, where the integer n can be calculated as:

$$n = \frac{1}{\Delta t \cdot \Delta f}$$

   In our case, most traces were recorded over a delay range with 500 fs in 1 fs steps and a wavelength region between 350 nm and 420 nm in steps of roughly 0.21 nm. Meanwhile, the rescaled trace has approximately three times the temporal and spectral step size, which corresponds to a trace resolution of 251x251 points, which is a good compromise between resolution and retrieval calculation speed.

5. SHG-FROG retrieval algorithm

For the retrieval of the time resolved pulse, a method called principal components generalized projection algorithm (PCGPA) was used. For our implementation, we mostly followed a description of Daniel Kane, one of the two inventors of the FROG method [3]. The PCGPA iteratively modifies the signal field, which is created by the SHG-process, until it fulfills both boundary conditions. These two conditions are:

1. The amplitude of the signal field has to match the measured FROG trace:

$$\mathrm{I_{FROG}} \sim \left|\mathrm{E_{sig}}(\omega, \tau)\right|^2$$

2. The signal field has to be created from the underlying SHG-process:

$$\mathrm{E_{sig}}(t, \tau) = \mathrm{E}(t) \cdot \mathrm{E}(t - \tau)$$

The first condition is enforced by replacing the signal field's amplitude with the root of the measured FROG trace in the frequency domain, while the second condition is enforced by decomposing the signal field in the time domain into a pulse field P(t) and gate field G(t), which correspond to the delayed and non-delayed field contributions of the signal field ($E_{sig}(t,\tau) = P(t) \cdot G(t - \tau)$) and then recombining them. For the decomposition, the signal field matrix elements are first rearranged into an outer product O of the pulse and gate field, which are then calculated as the eigenvalues of OO^T and O^TO using the singular value decomposition (SVD) or the power method. Afterward, the new signal field is calculated in a symmetric way, which ensures the equality of the pulse gate field in the long run:

$$\mathrm{E_{sig,new}}(t, \tau) = \mathrm{P}(t) \cdot \mathrm{G}(t - \tau) + G(t) \cdot \mathrm{P}(t - \tau)$$

For the initialization of the retrieval algorithm, we use an idea of Rana Jafari and Rick Trebino, where the spectrum of the pulse is deducted from the frequency marginal of the trace in a direct fashion [4], which only leaves the spectral phases as degrees of freedom, which are initialized randomly. This initial pulse is then used as the first guess for the pulse and gate field. After a sufficient amount of iterations, the pulse field can be extracted. It is also possible to monitor the progress of the retrieval process by comparing the retrieved trace with the target trace. Here, we performed the retrieval for all our measurements with twelve different initializations over 500 iterations and then choose the pulse with the best retrieved trace. A comprehensive overview of the PCGPA iteration cycle is sketched in Figure S7.

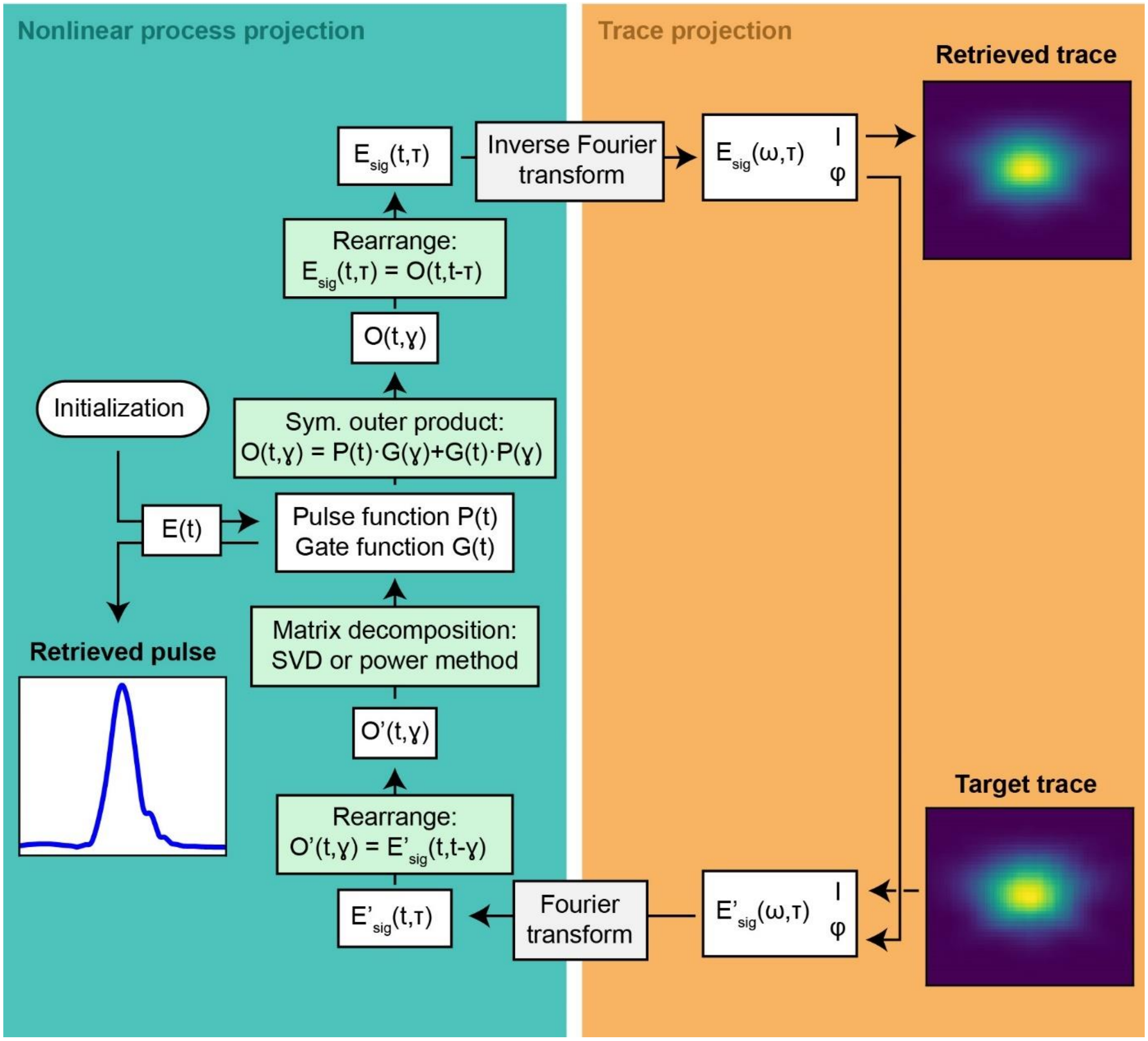

Figure S7: Iteration cycle of the principle component generalized projection algorithm (PCGPA), which is used for the pulse retrieval. In the algorithm, the signal field is projected on the two external boundary conditions, which are the creation of the FROG trace in the frequency space (orange box) and an underlying SHG-process in the time domain (turquoise box).

6. Measured SHG-FROG traces

In addition to the presented FROG traces in the main manuscript, we show here the originally measured (raw) traces together with the preprocessed and retrieved traces for different dispersion values of the used laser pulses (determined by the position setting of the external prism compressor).

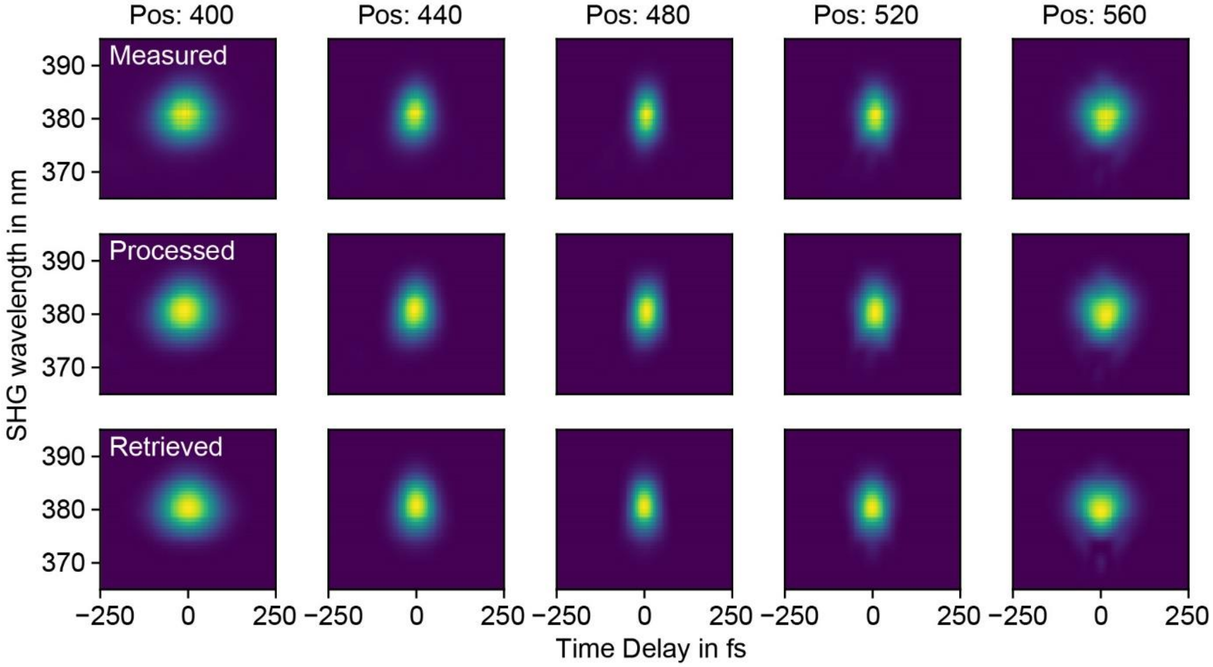

Figure S8. SHG-FROG traces for the reference measurement over various pulse compressor positions.

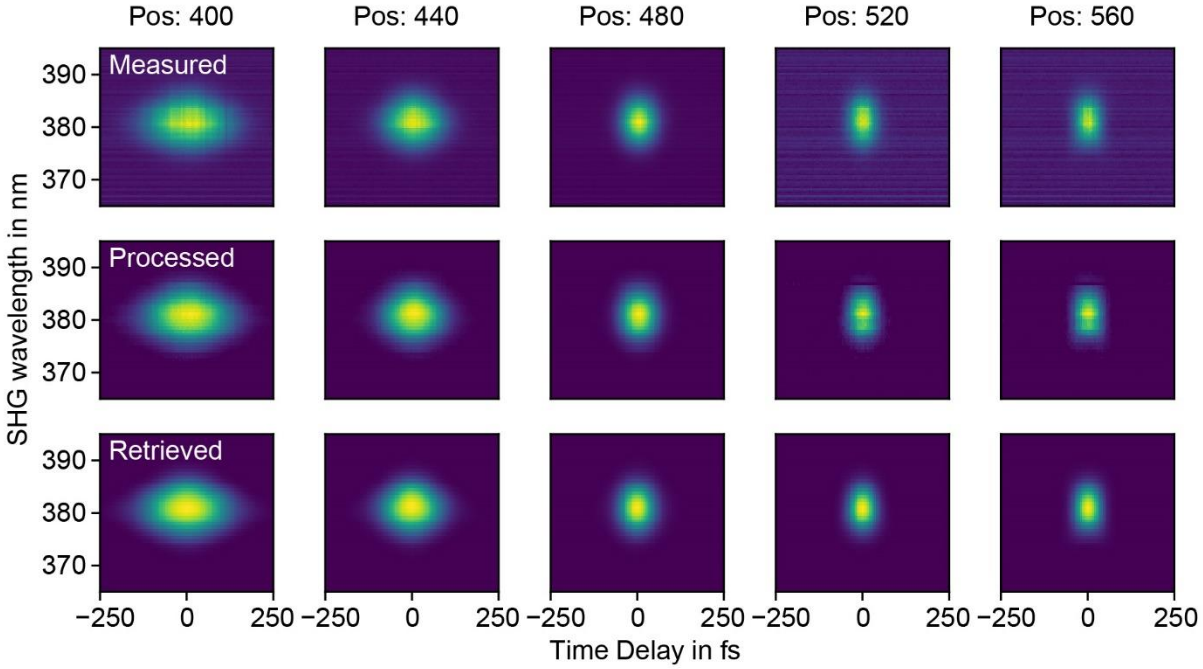

Figure S9: SHG-FROG traces for the uncompensated phase encoding over various pulse compressor positions.

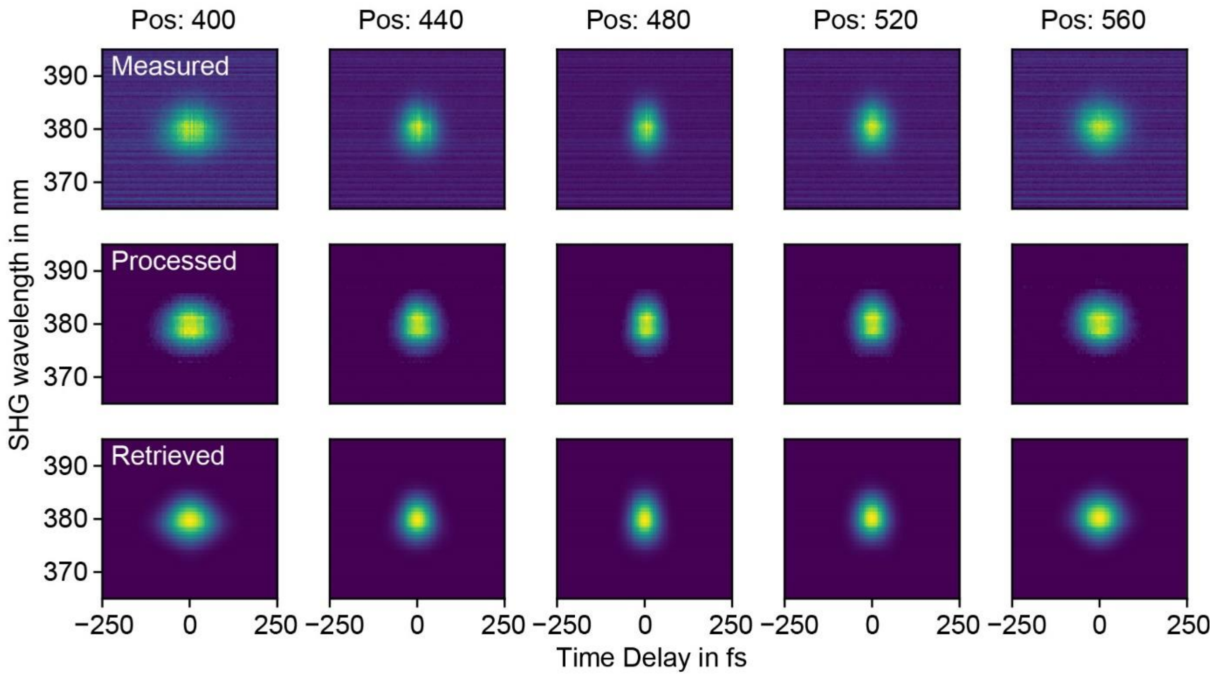

Figure S10: FROG traces for the compensated phase encoding over various pulse compressor positions.

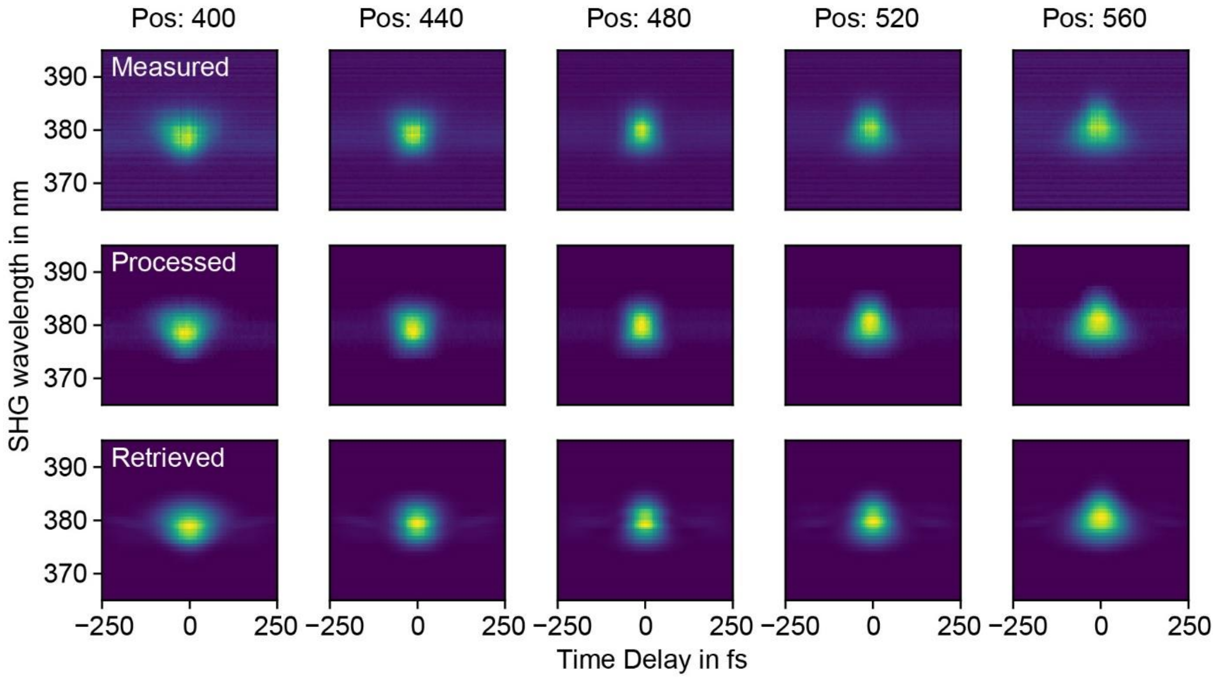

Figure S11: FROG traces for the 30k fs^3 TOD phase encoding over various pulse compressor positions.

References

1. Johnson, P.B. and R.W. Christy, *Optical Constants of the Noble Metals.* Physical Review B, 1972. **6**(12): p. 4370-4379.
2. Doost, M.B., *Resonant-state-expansion Born approximation for waveguides with dispersion.* Physical Review A, 2016. **93**(2): p. 023835.
3. Kane, D.J., *Principal components generalized projections: a review [Invited].* Journal of the Optical Society of America B-Optical Physics, 2008. **25**(6): p. A120-A132.
4. Jafari, R., T. Jones, and R. Trebino, *100% reliable algorithm for second-harmonic-generation frequency-resolved optical gating.* Optics Express, 2019. **27**(3): p. 2112-2124.
